# Electrochemical Interactions of Titanium and Cobalt–Chromium–Molybdenum Alloy in Different Solutions

**DOI:** 10.3390/ma19020367

**Published:** 2026-01-16

**Authors:** Anja Ivica, Matea Nimac, Ivica Pelivan, Matija Roglić, Tomislav Kovačević, Mario Cifrek, Jurica Matijević

**Affiliations:** 1 University of Zagreb School of Dental Medicine, Gundulićeva 5, 10000 Zagreb, Croatia; aivica@sfzg.unizg.hr (A.I.); matijevic@sfzg.unizg.hr (J.M.); 2 Department of Endodontics and Restorative Dentistry, Gundulićeva 5, 10000 Zagreb, Croatia; 3 Private Dental Clinic, Kranjčevićeva ulica 47, 10000 Zagreb, Croatia; matea.nimac@gmail.com; 4 Department of Removable Prosthodontics, Gundulićeva 5, 10000 Zagreb, Croatia; 5 University of Zagreb Faculty of Electrical Engineering and Computing, Unska 3, 10000 Zagreb, Croatia; matija.roglic@fer.unizg.hr (M.R.); tomislav.kovacevic@fer.unizg.hr (T.K.); mario.cifrek@fer.unizg.hr (M.C.)

**Keywords:** CoCrMo, CP4 titanium, BIACOM TiMg composite, electrochemical Interaction

## Abstract

Pure titanium (Ti) and its alloys are the gold standard for dental implants because a stable titanium dioxide passive film provides excellent corrosion resistance in physiological environments. In this study, we aimed to examine electrochemical interactions between Ti and cobalt–chromium–molybdenum alloy (CoCrMo), and between a novel Ti–magnesium composite (BIACOM TiMg) and CoCrMo, when immersed in everyday solutions representing beverage or oral hygiene exposure. Test solutions included Coca-Cola^®^, lemon juice, Elmex^®^ fluoride gel, Listerine^®^ Cool Mint, and Sensodyne^®^ fluoride paste. Immersion experiments paired Ti sticks with CoCrMo sticks and, separately, BIACOM TiMg with CoCrMo sticks, with three measurements per configuration. When galvanically coupled with CoCrMo, immersion in Coca-Cola produced galvanic potential differences of ~983 mV for the BIACOM TiMg-CoCrMo couple and 830 mV for the commercially pure grade 4 (CP4) Ti-CoCrMo couple, indicating significant electrochemical instability. Both materials showed significant potential increases in Elmex fluoride gel. Listerine Cool Mint and Sensodyne fluoride exposure produced electrochemical interactions exceeding 200 mV. Significant differences in corrosion stability were observed between CP4 Ti and BIACOM TiMg. These findings indicate that material pairing and electrolyte environment significantly influence galvanic behavior, with the Ti-Mg composite showing greater susceptibility than CP4 Ti, informing dental/biomedical material selection in oral environments.

## 1. Introduction

Metals and their alloys are widely used in dentistry due to their favorable properties [1]. Titanium (Ti) is the most commonly used material in implant prosthodontics because of its superior osseointegration and passivation properties [2]. In prosthetic treatment, Ti is used for abutments, similar to implant fixtures. However, the final restoration is fabricated from a different material—usually cobalt–chromium–molybdenum (CoCrMo)—which offers superior castability and must be considered because differences in standard electrode potentials influence corrosion mechanisms [3]. Galvanic corrosion commonly occurs in the oral cavity when a Ti dental implant is coupled with a suprastructure made of a different metal alloy, creating electrochemical potential differences in the presence of an electrolyte, such as saliva or other bodily fluids [4]. Acidic conditions and high fluoride concentrations, commonly found in oral care products such as toothpastes and fluoride gels, further increase the risk of corrosion [5]. This process leads to the formation of a galvanic cell, in which one metal acts as the anode and the other as the cathode [6]. A significant challenge affecting the longevity and success of Ti-based dental implants is material corrosion, particularly galvanic and pitting corrosion [7].

Previous studies have shown a correlation between corrosion in galvanic systems composed of a Ti implant and a superstructure made of a different alloy and the subsequent failure of implant prosthetic therapy [8]. Galvanic corrosion in implant–superstructure systems can release corrosion products from alloy components, which may cause biological effects such as local inflammatory responses, allergic reactions, or systemic toxicity [9]. Titanium debris has been observed in some clinical cases to induce immune signaling that contributes to the development of type I and type IV hypersensitivity responses [10]. In galvanic couples involving Ti-based materials and CoCrMo alloy, the Ti-based component acts as the anodic material and therefore corrodes more rapidly, leading to structural weakening over time and compromising the integrity, durability, and functionality of the dental implant system [11]. In addition, corrosion in the oral cavity may arise from direct interactions between metallic materials and oral environmental factors, such as saliva composition, acidic pH, and fluoride-containing agents, which can further destabilize passive oxide layers [12]. Furthermore, the electrical current generated by galvanic coupling may influence bone metabolism, potentially leading to bone resorption at the implant site [13]. In this context, interactions between Ti-based materials and CoCrMo alloys, particularly when used in close proximity or as components of multi-component prosthetic systems, warrant careful investigation. The galvanic potential difference between alloy pairings can influence the magnitude of electrochemical currents and dictate corrosion behavior. Previous studies have demonstrated that coupling Ti and CoCrMo can produce measurable galvanic currents under specific conditions [6].

In recent years, novel composite materials, such as Ti–Magnesium (Ti-Mg) composites (BIACOM TiMg), have emerged to combine favorable mechanical and biological properties with lower stiffness or improved bioactivity [14]. The BIACOM TiMg composite is designed for controlled biodegradation and intended for temporary clinical applications (service life of weeks to months) that require short-term biological integration rather than permanent implant fixation. However, when such biodegradable materials are galvanically coupled with permanent metallic components, such as CoCrMo suprastructures, electrochemical interactions during the intended service period require characterization [15]. However, the electrochemical stability of BIACOM TiMG composites in everyday fluid environments, such as common beverages or oral hygiene solutions, remains unclear. Therefore, in this study, we aimed to examine whether electrochemical interactions occur between Ti and CoCrMo alloy, and between a Ti–Mg composite (BIACOM TiMg) and CoCrMo alloy, when immersed in a range of everyday solutions representing beverage or oral hygiene exposure. We sought to quantify galvanic and corrosion-related behavior and compare the relative stability of these material pairings by performing controlled immersion experiments and measuring electrical potentials in realistic fluid media, including Coca-Cola^®^, lemon juice, fluoride gel, mouth rinse, and fluoride dental paste. These findings have implications for selecting dental and biomedical material combinations that are in contact or close proximity within the oral environment by highlighting differences in corrosion susceptibility under conditions that mimic real-world exposure.

## 2. Materials and Methods

### 2.1. Sample Preparation

The Research Ethics Committee of the Dental School, University of Zagreb, approved the study (approval number: 05-PA-30-VI-4/2019).

In this study, three distinct specimen types were employed:

- A cast rod of the CoCrMo alloy (commercial product: Heraenium P, Kulzer Mitsui Chemicals Group, Hanau, Germany) with a diameter of 1 mm and a length of 18 mm. The alloy composition was 59.0 wt% Co, 25.0 wt% Cr, 4.0 wt% Mo, 10.0 wt% tungsten (W), 1.0 wt% silicon (Si), 0.8 wt% manganese (Mn), and 0.2 wt% nitrogen (N). It is compliant with all the requirements of EN ISO 9693 [16] and EN ISO 22674 [17].
- A rod of commercially pure Ti (CP4 grade) and a metal composite of CP-Ti and magnesium (BIACOM TiMg) specimens were produced in comparable rod-shaped geometry, consistent with standard in vitro galvanic testing practices:
  ○ CP4 Ti with dimensions of 2 mm × 2 mm × 10 mm, produced by laser-sintering powder metallurgy at the Faculty of Mechanical Engineering and Naval Architecture, University of Zagreb.
  ○ BIACOM TiMg with a diameter of 5 mm and a length of 11 mm, manufactured by low-temperature cold extrusion of Ti and Mg powders at the Faculty of Mechanical Engineering and Naval Architecture, University of Zagreb.

The chemical compositions of the investigated alloys were determined by X-ray fluorescence spectroscopy using an Olympus Innov-X system (Woburn, MA, USA).

### 2.2. Electrode Assembly

**Figure 1:**
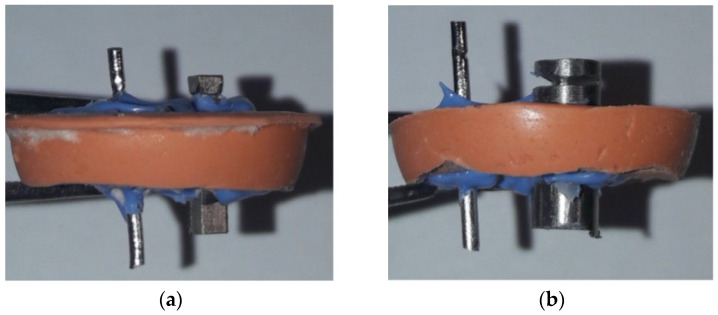
The electrodes were secured using a silicone holder made of additional silicone, and the gap was additionally sealed with a very low-viscosity addition silicone: (**a**) CoCrMo alloy and Ti rod (**b**) CoCrMo alloy and Ti alloy.

In the first measurement phase, the CP4 Ti rod served as one electrode, while the CoCrMo rod served as the counter-electrode. In the second phase, the BIACOM TiMg composite rod served as one electrode and was again paired with the CoCrMo rod as the counter-electrode. The electrodes were mounted in a silicone clamp fabricated from addition-curing, putty-consistency silicone (3M ESPE Express STD firmer set putty, 3M Deutschland, Neuss, Germany). The inter-electrode spacing was sealed with a low-viscosity, addition-curing silicone (3M ESPE Express Light Body fast set, 3M ESPE, St. Paul, MN, USA) to isolate the exposed surfaces and define the electrolyte interface (Figure 1).

After each immersion measurement, the electrodes were rinsed in tap water, dried, immersed in a hypotonic solution (Aqua pro injectione, HZTM, Zagreb, Croatia), and dried with isopropanol (Kontakt IPA plus, AG Termopasty, Sokoły, Poland). Finally, the rods were allowed to air-dry completely before the next measurement series.

### 2.3. Solutions

**Figure 2:**
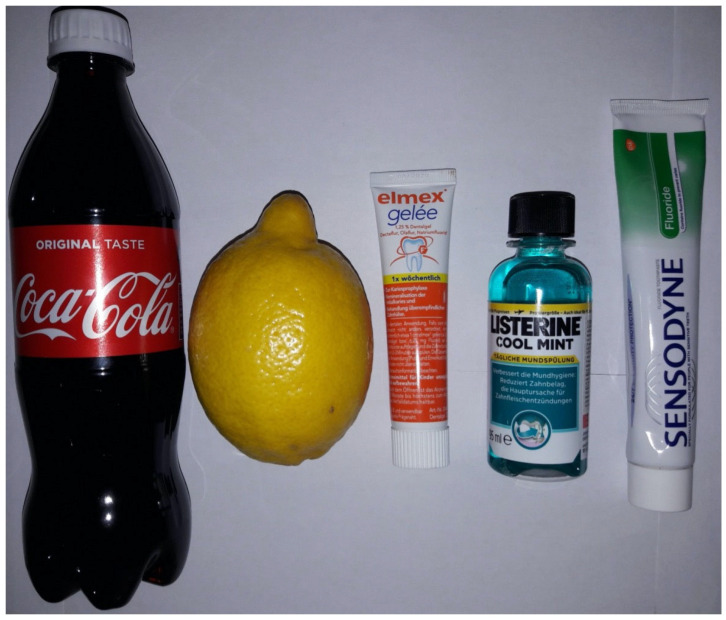
Different test solutions used in the immersion experiments: Coca-Cola^®^, freshly squeezed lemon juice, Elmex^®^ fluoride gel, Listerine^®^ Cool Mint mouth rinse, and Sensodyne^®^ Fluoride toothpaste.

The solutions used in the immersion experiments were as follows (Figure 2):

- Coca-Cola^®^ (Coca-Cola Hrvatska, Zagreb, Croatia).
- Freshly squeezed lemon juice.
- Elmex^®^ fluoride gel (CP GABA GmbH, Hamburg, Germany).
- Listerine^®^ Cool Mint mouth rinse (Johnson & Johnson, New Brunswick, NJ, USA).
- Sensodyne^®^ fluoride toothpaste (GSK, London, UK).

Each solution was placed in excess volume in a silicone vessel to ensure complete submersion of the samples during measurement.

### 2.4. Measurement of Electrochemical Potentials

All measurements were conducted at the Department of Electronic Systems and Information Processing, Faculty of Electrical Engineering and Computing, University of Zagreb. Sample geometries were employed to facilitate controlled electrochemical measurements under laboratory conditions. Rod-shaped specimens were produced to enable consistent two-electrode galvanic coupling (CP4 Ti: 2 mm × 2 mm × 10 mm, CoCrMo: 1 mm diameter × 18 mm length, BIACOM TiMg: 5 mm diameter × 11 mm length). These configurations are consistent with standard in vitro corrosion testing practices. The open-circuit potential (OCP) values and potential differences measured between material pairs are independent of sample dimensions and reflect the intrinsic electrochemical properties of the tested material combinations. However, absolute galvanic current magnitudes and corrosion rates would scale proportionally with electrode surface area in clinical configurations. For each immersion solution, three independent measurements, each lasting 3 min, were performed. The experiment consisted of two phases. In the first phase, one electrode was a commercially pure Ti (CP4) rod and the other was a CoCrMo rod. In the second phase, one electrode was the BIACOM TiMg composite rod and the other was the CoCrMo rod. The electrodes were connected via electrical clamps to a digital multimeter (Fluke 45, Fluke Europe, Eindhoven, The Netherlands), which transferred sampled data to a personal computer. A custom graphical interface developed in MATLAB (R2025b, MathWorks, Germany) was used to visualize, analyze, and process the recorded potential signals.

Notably, the galvanic potential differences measured between coupled electrodes are distinct from the individual OCP measurements described in Section 3.2. Individual OCP measurements characterize the intrinsic electrochemical potential of each isolated metal relative to a standard silver/silver chloride reference electrode. In contrast, the galvanic coupling measurements presented here quantify the potential difference that develops when two different metals are in direct electrical contact within the same electrolyte solution, forming a galvanic cell. These galvanic coupling measurements directly reflect the driving force for corrosion in multi-material dental implant systems.

The exposed surface areas of the coupled specimens were defined by the silicone holder and sealing procedure, which limited electrolyte contact to comparable regions of each electrode. Galvanic corrosion rates depend on the anode-to-cathode surface area ratio; however, in this study, we focused on measuring galvanic potential differences rather than galvanic current densities. Therefore, the results primarily reflect differences in electrochemical interaction and the driving force for galvanic corrosion rather than quantitative corrosion rates.

Each run lasted 3 min because the galvanic potential difference exhibited the most significant transient change immediately after immersion, reflecting rapid establishment of the metal–electrolyte interface and early passive-film response in the tested media. Longer-term stabilization was also observed qualitatively, with the galvanic potential gradually decaying from the initial peak and approaching a stable value over approximately 24 h. This time-dependent behavior warrants systematic investigation in dedicated long-term immersion studies.

### 2.5. Statistical Data Processing

Voltage levels were recorded every second and saved as .mat files using a script written in MATLAB. The maximum and minimum values were extracted from the 180 collected data points. When selecting the minimum value, the first few seconds were excluded to avoid during electrode immersion in the electrolyte. The voltage range was calculated as the difference between the maximum and minimum values to describe the dynamic behavior of each electrolyte:

$$∆U=U_{max}-U_{min}$$

The geometric mean was calculated for all three parameters to obtain an average value characteristic of each electrolyte and electrode pair. The geometric mean was calculated using the following equation:

$$U=\sqrt[{3}]{U_{1}\times U_{2}\times U_{3}}$$

## 3. Results

### 3.1. X-Ray Fluorescence Spectroscopy

The chemical compositions of the investigated alloys were determined by X-ray fluorescence spectroscopy (Olympus Innov-X system). The Ti-based material consisted predominantly of Ti, with aluminum (Al) and vanadium (V) as the principal alloying elements, corresponding to a Ti–6Al–4V-type alloy. Iron (Fe) was present as a minor impurity (Table 1). The Co-based alloy was composed primarily of Co and Cr, with additions of Mo and W, which is characteristic of CoCrMo alloys commonly used in biomedical and dental applications (Table 2). Phosphorus (P) was present in trace amounts (<0.1%).

### 3.2. Individual OCP Measurements vs. Reference Electrode

**Figure 3:**
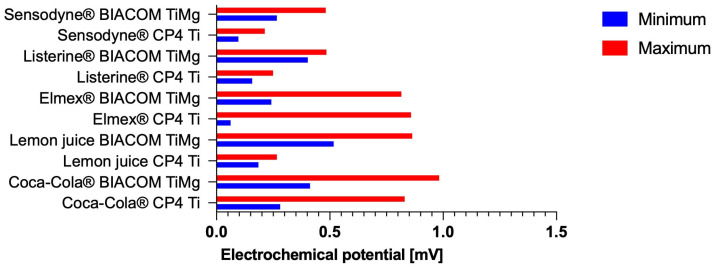
Galvanic potential differences (millivolts) between coupled metal pairs measured in different test solutions. Data represent the average minimum and maximum values from three independent measurements for each material pairing (Ti-CoCrMo or BIACOM TiMg-CoCrMo) and electrolyte.

OCP measurements were performed to establish the intrinsic electrochemical nobility of each alloy before galvanic coupling and to assess the thermodynamic driving force for electrochemical interactions between Ti-based implants and CoCrMo alloys in oral-relevant environments (Figure 3). The OCP of individual Ti and CoCrMo specimens was recorded using a potentiostat controlled by 352 SoftCorr III software (version 3.5.2; Eg&G Instruments, Boston, MA, USA), with the potential measured relative to a reference electrode under open-circuit conditions. Measurements were conducted for 1000 s to ensure stabilization of surface passive films, particularly the titanium dioxide (TiO_2_) layer on Ti and the Cr-rich oxide on CoCrMo. The stabilized OCP values for the Ti-based and CoCrMo alloys served as a baseline for interpreting subsequent galvanic coupling experiments and corrosion behavior in the tested electrolytes (Table 3).

**Figure 4:**
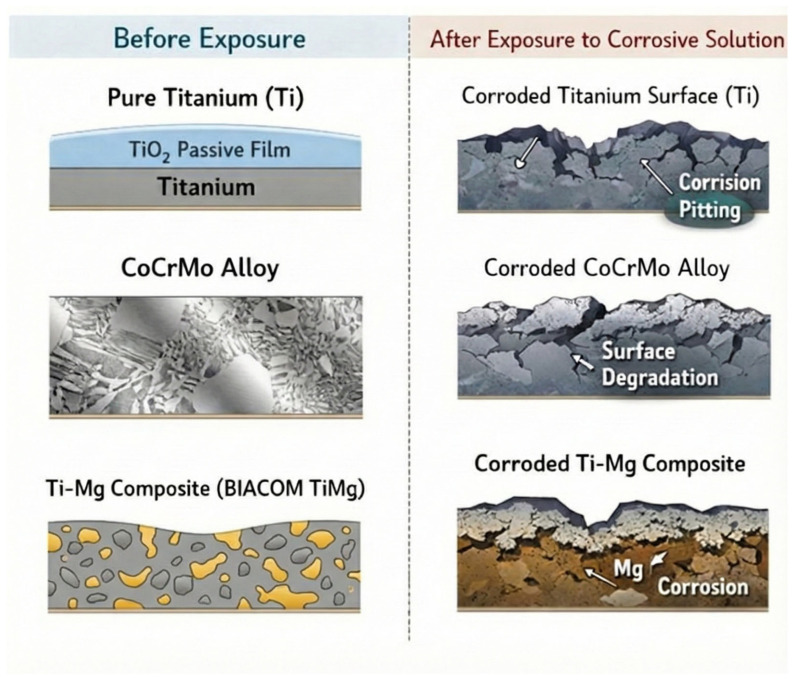
Schematic representation of the material surfaces before and after exposure to corrosive solutions. The drawings illustrate the qualitative surface condition of CP4 Ti and BIACOM TiMg composite galvanically coupled with CoCrMo. Before immersion, an intact passive oxide layer is shown. After exposure, the schematics depict representative surface changes inferred from electrochemical measurements, including partial passive film degradation, enhanced galvanic interaction, and increased corrosion activity in aggressive media. The illustrations are conceptual and do not represent actual surface images.

The electrochemical results are supported by a conceptual schematic illustration of the material surfaces before and after immersion in the corrosive solutions (Figure 4), which qualitatively summarizes the inferred surface changes and galvanic interactions.

## 4. Discussion

In this study, we demonstrate that the electrochemical behavior of Ti-based materials in contact with CoCrMo strongly depends on the surrounding electrolyte environment. Across all tested solutions, CP4 Ti and the BIACOM TiMg composite exhibited measurable galvanic interactions with CoCrMo, with the most significant potential increases observed in Coca-Cola and Elmex fluoride gel.

Galvanic corrosion may occur when two metals with different electrode potentials are present in the oral cavity [18]. The presence of an electrolyte is required for electrochemical activity, and in the oral environment this role is fulfilled by saliva and surrounding soft and hard tissues. Electrons flow spontaneously from the anode to the cathode, generating a potential difference at the metal-electrolyte interface. The metal with the lower electrode potential undergoes preferential dissolution and therefore serves as the anode, while the more noble metal acts as the cathode. Oxidation, also referred to as the anodic reaction, occurs at the anode, during which metal atoms are converted into ions and electrons. Concurrently, reduction (the cathodic reaction) occurs at the cathode through the consumption of electrons [19].

Notably, galvanic corrosion rates depend on the anode-to-cathode surface area ratio. The exposed areas were constrained to similar dimensions; however, we did not quantify current density or material loss. Consequently, the results should be interpreted as comparative indicators of galvanic driving force rather than absolute corrosion severity.

In this study, we captured the early-time galvanic response (first minutes) after immersion, when the largest potential changes occurred. Because passive-film evolution and corrosion processes are time-dependent, long-term immersion and complementary electrochemical methods (e.g., extended OCP monitoring and/or electrochemical impedance spectroscopy [EIS]) are needed to fully characterize stabilization and drift over hours to days.

Corrosion dynamics depend on multiple factors. Beyond the intrinsic properties of a metal, corrosion depends on its interaction with the surrounding environment [6]. In addition, conditions in the oral cavity vary significantly. When eating, food temperature may range from 0 °C to 70 °C, pH may vary from approximately 2 to 11, and electrolyte concentration can change accordingly [20]. Moreover, gas and oxygen diffusion at the surface of removable prosthodontic devices may contribute to electrochemical degradation processes similar to atmospheric corrosion, which refers to material degradation caused by exposed to air, oxygen, water vapor, and pollutants. This mechanism can occur on prosthetic appliances exposed to the environment rather than fully immersed in a liquid electrolyte [21]. In addition to corrosion, material dissolution may also occur, which represents the intended degradation mechanism of the BIACOM TiMg composite [22].

Our results indicate that immersion in Coca-Cola produced a significant increase in electrochemical potential. Coca-Cola induced significant electrochemical instability, with maximum values of ~829 mV for CP4 Ti and ~982 mV for the BIACOM TiMg composite. The pH of Coca-Cola was approximately 2.37. A similarly high maximum potential of ~863 mV was observed for the BIACOM TiMg composite immersed in freshly squeezed lemon juice. Numerous studies measuring potential differences between Ti implants and overlying suprastructures in artificial saliva have shown that pH variability and temperature oscillations further accelerate corrosion [15,23].

Similarly, the results demonstrate increased electrochemical interaction when Elmex fluoride gel (12,500 ppm fluoride, pH ~ 4.8) was used as the electrolyte. Fluoride prophylaxis is clinically beneficial; however, it has been shown to induce corrosion instability in Ti at subneutral pH values [24]. High fluoride concentrations combined with low pH have been identified as detrimental to Ti corrosion resistance; for example, previously reported threshold pH values were ~4.0 at 0.05% sodium fluoride (NaF) and 4.3 at 0.1% NaF [25].

The negative impact of fluoride on Ti passivity observed in our study using Elmex fluoride gel is supported by recent research on fluoride-containing oral care products. Slama et al. performed electrochemical assessments of innovative Ti-based high-entropy alloys in artificial saliva containing fluoride, directly addressing environmental conditions relevant to dental applications [26]. Their findings indicated that fluoride ions significantly influenced the corrosion behavior of Ti-based materials in saliva-like electrolytes, with the degree of passivity destabilization depending on both fluoride concentration and alloy composition. The authors also highlighted that fluoride can undermine the protective TiO_2_ layer through complexation processes that generate soluble Ti-fluoride species, particularly in acidic environments where the passive film is already compromised. This mechanism elucidates the significant potential increases observed with Elmex fluoride gel (12,500 ppm fluoride, pH ~4.8), as the combination of elevated fluoride concentration and acidic pH creates favorable conditions for passive film degradation.

Fluoride-induced corrosion has clinical consequences that extend beyond material degradation. When Ti implants are combined with different metals, such as CoCrMo, in the presence of fluoride, weakening of the passive Ti coating may accelerate galvanic corrosion and increase ion release from both materials. The potential shifts of approximately 800 mV observed in our investigation with fluoride-containing media represent a strong electrochemical force for metal dissolution. These findings suggest that individuals with Ti implants connected to CoCrMo prosthetic components should receive guidance on selecting oral care products, particularly those with high fluoride concentrations or low pH. Fluoride delivery methods with neutral pH or lower fluoride concentrations may better preserve the electrochemical stability of multi-material implant systems.

Electrochemical interactions exceeding 200 mV were recorded with Listerine Cool Mint mouth rinse and Sensodyne fluoride toothpaste. Under identical solution conditions, the BIACOM TiMg specimen developed nearly twice the potential observed for CP4 Ti. In the same solutions, potential values were significantly lower for CP4 Ti. Many Ti-based alloys exhibit superior mechanical properties compared with pure Ti; however, they often demonstrate reduced chemical stability [27]. Furthermore, given the designed solubility of Mg in the metal composite, the increased electrochemical activity of the BIACOM TiMg sample is plausible [6].

The distinct electrochemical responses observed between CP4 Ti and the BIACOM TiMg composite in this study are consistent with previous reports of alloy-dependent corrosion behavior in oral environments. Turkina et al. investigated the effects of commonly used dry-mouth products on the corrosion resistance of prevalent dental alloys, including Ti-6Al-4V and CoCr alloys [28]. Their results showed that different dental care products altered OCPs, corrosion potentials, and corrosion currents in a material-specific manner. They reported pitting corrosion of CoCr alloys, which led to Co and Cr ion release in some products, whereas Ti alloys remained comparatively passive. The behavior observed for this product and alloy supports our finding that the electrochemical stability of Ti-based materials varies significantly depending on material composition and the specific electrolyte environment encountered in clinical practice.

The increased electrochemical activity of the BIACOM TiMg composite relative to CP4 Ti may be influenced by the composition and stability of the passive oxide layer. Recent studies on innovative Ti alloys have demonstrated that alloying elements significantly affect passive film properties and repassivation kinetics. Teixeira et al. assessed Ti-15 zirconium (Zr) and Ti-15Zr-5Mo biomaterial alloys in phosphate-buffered saline and discovered that these alloys exhibited enhanced passive behavior and superior passive film recovery after perturbation compared with commercially pure Grade 4 Ti [29]. Electrochemical impedance spectroscopy analysis demonstrated higher polarization resistance in the alloyed materials, indicating improved corrosion resistance and passive film stability compared with commercially pure Ti.

In contrast, the addition of Mg to the BIACOM composite serves a different purpose—controlled biodegradation. However, the principle that alloying elements alter passive film stability and electrochemical response remains applicable. The engineered solubility of Mg likely weakens the protective ability of the oxide layer, which may explain the higher potentials observed in the galvanic coupling tests. The elevated electrochemical activity observed in the BIACOM TiMg composite reflects its intentional design for controlled biodegradation. It is important to emphasize that BIACOM TiMg is not intended to replace CP4 Ti in permanent implant fixtures; rather, it is designed as a bioabsorbable material for temporary orthopedic or periodontal scaffold applications. The enhanced galvanic activity with CoCrMo observed in this study, particularly the extreme potentials exceeding 800 mV in Coca-Cola and 200 mV in fluoride-containing products, may exceed the controlled degradation rate intended for this composite and result in unpredictable dissolution kinetics. Therefore, if BIACOM TiMg is used clinically, its application should be limited to temporary scaffold systems with compatible temporary components rather than with permanent suprastructure materials intended for long-term use.

Ti and its alloys form a protective passive oxide layer when in contact with biological media and metallic structures. This oxide layer typically consists of TiO_2_ in various polymorphic forms, including rutile and anatase, as well as hydrated oxide species [30]. Under neutral pH and physiological conditions, the passive layer confers excellent biocompatibility and bioinert behavior. However, the stability of this layer is highly environment-dependent and can be compromised by acidic pH, elevated fluoride concentrations, and high electrochemical potentials, as demonstrated in this study. The thickness and composition of the oxide film, rather than the underlying alloy composition alone, are crucial determinants of the protective capacity of the passive layer against toxic ion release [31]. The passive layer slows corrosion; however, it does not prevent it and can undergo continuous repassivation depending on environmental conditions [32].

In this study, we focused on electrochemical potential measurements to evaluate galvanic interactions between Ti-based materials and CoCrMo alloys in oral-relevant environments. Detailed characterization of surface morphology, chemical composition, and passive oxide layer properties, including TiO_2_ thickness and composition, was not performed. Such analyses would provide additional insight into corrosion and degradation mechanisms, particularly passive film evolution, selective dissolution, and surface modification; however, their absence does not affect the comparative assessment of galvanic driving forces reported here. Comprehensive surface and oxide-layer characterization will be addressed in future investigations.

The passive film composed of TiO_2_ is highly sensitive to environmental changes and surface treatments. Recent studies have elucidated how manufacturing techniques and ambient conditions influence TiO_2_ layer properties and, consequently, corrosion resistance. Igual-Muñoz et al. examined the effects of various sterilization techniques on the surface chemistry and electrochemical properties of biomedical alloys, specifically Ti and CoCrMo [33]. They discovered that autoclave sterilization increased Cr concentration in the passive coating of CoCrMo alloys, thereby enhancing corrosion resistance. In contrast, combined autoclave and ultraviolet light treatment accelerated corrosion kinetics in Ti alloys by increasing oxygen reduction kinetics at the surface. This finding is particularly relevant to our study, as it demonstrates that even routine clinical processing can alter passive film properties and modify the electrochemical response of Ti-based materials upon subsequent exposure to oral fluids.

The phase composition of the TiO_2_ layer also affects ion release and long-term stability. Javadi et al. investigated metal ion release from a plasma electrolytic oxidation (PEO)-coated Ti-6Al-4V alloy fabricated by direct metal laser sintering [34]. Their study showed that PEO coatings composed of anatase and rutile phases retained approximately 98–99% of oxidized Ti within the passive layer under normal conditions. However, once present, coatings could also become the primary source of ion release, with coating composition and microstructure determining whether the oxide layer functioned as a protective barrier or a source of soluble species. The dual role of oxide layers as both protective coatings and potential ion sources complicates prediction of corrosion progression over time in clinical settings. In the context of this study, differences in the quality of the native oxide layer between CP4 Ti and the BIACOM TiMg composite may explain their differing electrochemical responses when coupled with CoCrMo.

A properly processed surface is a prerequisite for forming a stable passive film [35]. Surface morphology analysis before and after corrosion testing provides direct evidence of localized degradation and topographical changes that electrochemical measurements alone cannot capture, thereby improving understanding of how corrosion affects surface integrity. We did not examine surface morphology; however, correlating electrochemical results with direct morphological evidence would strengthen the mechanistic interpretation of corrosion phenomena. Therefore, future work will include scanning electron microscopy or atomic force microscopy, along with elemental mapping before and after exposure to the test environment, to more comprehensively characterize corrosion progression and relate morphological changes to the observed electrochemical responses. Moreover, interpretation of corrosion mechanisms based on the time evolution of OCP curves was beyond the scope of the present study and will be addressed in future investigations.

In this study, we focused on potential measurements; however, subsequent investigations of CoCrMo alloy behavior provide a mechanistic framework for the observed galvanic interactions. Yilmazer et al. examined the corrosion and tribocorrosion characteristics of CoCrMo alloys subjected to various processing techniques, including high-pressure torsion and solution treatment [34]. Their study showed that CoCrMo is generally corrosion resistant in phosphate-buffered saline but is highly susceptible to tribocorrosion under sliding conditions. This mechanical-electrochemical synergy is particularly relevant in the oral environment, where prosthetic components may experience slight micromovements, occlusal loading, and abrasive contact with food particles or opposing teeth. Mechanical disruption of the CoCrMo passive layer can lead to localized breakdown, transient increases in anodic activity, and, when coupled with Ti, amplification of galvanic reactions.

The electrochemical behavior of CoCrMo components in galvanic couples is also influenced by manufacturing methods and microstructure. The same study showed that solution-treated CoCrMo samples experienced greater material loss under tribocorrosion conditions than cast or high-pressure torsion-processed materials, despite having similar static corrosion resistance [36]. These findings indicate that static immersion tests alone cannot adequately predict the electrochemical stability of CoCrMo in multi-material dental systems. In clinical Ti-CoCrMo couples, mechanical factors, including prosthetic fit, load-bearing capacity, and interfacial micromotion, can significantly influence the extent and effects of galvanic corrosion. Future studies should integrate tribocorrosion testing techniques that replicate the complex mechanical-electrochemical environment of functional dental implant systems to yield more clinically relevant predictions of material degradation and ion release.

A key consideration for clinical translation is that electrochemical potentials are geometry-independent, whereas galvanic currents scale proportionally with electrode surface area. Therefore, the relative stability rankings observed in this study—CP4 Ti demonstrating greater stability than the BIACOM TiMg composite across most oral environments—are clinically relevant regardless of sample size. Similarly, identification of problematic electrolyte environments (Coca-Cola, Elmex fluoride gel with low pH and high fluoride concentration, Listerine, and Sensodyne) applies to full-scale implant components. However, quantitative prediction of ion release rates and corrosion magnitude in clinical-size implant abutments and crown suprastructures require future studies using component-sized specimens or validated mathematical scaling algorithms that account for geometric differences between laboratory models and clinical configurations.

Corrosion may also contribute to implant failure by compromising mechanical stability and the integrity of surrounding tissues. Release of metal ions or electrochemical by-products can cause aseptic loosening, bone resorption (osteolysis), or inflammatory responses mediated by macrophage activation and, in rare cases, potential neoplastic changes [37]. In one study, galvanic potentials exceeding 100 mV were observed in 84% of patients presenting with oral pathological changes and symptoms; the most common pathological finding was lichen planus, and reported symptoms included xerostomia and dysgeusia [35]. Normal oral electrical potentials between two metals, or between a metal and the mucosa, are approximately 100 mV, and potentials up to 200 mV typically do not provoke symptoms [38]. When patients report subjective symptoms such as burning or tingling upon contact with metals in the oral cavity (e.g., during eating), this is considered evidence of a galvanic cell [39]. In such cases, potentials exceeding 200 mV indicate the need for removal of the metal from the oral cavity.

This study has some limitations that warrant acknowledgement. First, the experiments were conducted under static laboratory conditions that do not fully replicate the complexity of the oral environment, where factors such as saliva composition, temperature fluctuations, enzymatic activity, and mechanical loading can influence corrosion behavior. Second, only short-term immersion tests were performed; therefore, long-term degradation kinetics and passive film stability over extended periods remain uncertain. Third, the analysis was focused solely on potential measurements. Complementary techniques, such as electrochemical impedance spectroscopy, surface morphology assessment, or ion-release analysis, were not performed and would provide a more comprehensive understanding of corrosion mechanisms. Finally, the limited sample size for each configuration restricts the statistical power of the findings.

Furthermore, the tested samples were smaller than actual clinical dental components to facilitate controlled electrochemical measurements under laboratory conditions. The measured open-circuit potentials and potential differences are independent of sample geometry; however, the surface area-to-volume ratios of the specimens differ from those of clinical components. Consequently, absolute galvanic current magnitudes and ion release kinetics may scale differently in full-size implant abutments and crown superstructures. Future studies using clinically scaled component geometries are therefore required to extrapolate these electrochemical potential findings to quantitative predictions of corrosion behavior in clinical use.

EIS was not employed in this study. EIS is a powerful tool for investigating corrosion kinetics and passive film properties of individual materials; however, its interpretation in galvanically coupled, dissimilar-metal systems immersed in complex electrolytes is challenging and requires extensive modeling. Accordingly, we focused on galvanic potential measurements as a direct and comparative indicator of electrochemical interaction under oral-relevant conditions. Future studies will incorporate EIS to provide a more detailed mechanistic understanding of corrosion processes.

## 5. Conclusions

In this study, our findings indicate that common beverages and oral hygiene products can significantly alter the galvanic behavior of Ti–CoCrMo and TiMg–CoCrMo pairs by affecting passive film stability. The significant potential shifts observed in low-pH and fluoride-containing environments indicate compromised corrosion resistance, particularly for the Ti–Mg composite. For permanent, long-term multi-material implant systems, CP4 Ti demonstrated superior electrochemical stability compared with the BIACOM TiMg composite. In contrast, BIACOM TiMg is designed for controlled biodegradation in temporary applications, with a service life of weeks to months rather than permanent fixation. The significant galvanic instability observed when BIACOM TiMg was coupled with CoCrMo, particularly in acidic beverages and fluoride-rich products, indicates that its electrochemical behavior may exceed the intended controlled degradation range, raising concerns regarding long-term pairing with permanent suprastructure components. Consequently, CP4 Ti appears more suitable for permanent multi-material implant systems, while BIACOM TiMg should be restricted to temporary scaffold applications involving compatible temporary or absorbable component pairings.

## Figures and Tables

**Table 1:** Elemental composition of the Ti-based alloy determined by X-ray fluorescence spectroscopy (wt%).

Element	Ti	Al	V	Fe
Wt%	90.3	5.3	4.2	0.2

**Table 2:** Elemental composition of the CoCrMo alloy determined by X-ray fluorescence spectroscopy (wt%).

Element	Co	Cr	Mo	W	Al	P
Wt%	60.5	23.2	4.7	5	6.6	<0.1%

**Table 3:** Summary of galvanic potential differences (mV) showing the range (maximum minus minimum values) for Ti-CoCrMo and BIACOM TiMg-CoCrMo couples in five test solutions.

	Sensodyne CP4 Ti	Sensodyne BIACOM TiMg	Lemon Juice CP4 Ti	Lemon Juice BIACOM TiMg	Elmex Gel CP4 Ti	Elmex Gel BIACOM TiMg	Listerine CP4 Ti	Listerine BIACOM TiMg	Coca Cola CP4 Ti	Coca Cola BIACOM TiMg
Range [mV]	0.104	0.206	0.141	0.328	0.736	0.566	0.09	0.078	0.501	0.57

## Data Availability

The original contributions presented in this study are included in the article. Further inquiries can be directed to the corresponding author.

## Abbreviations

The following abbreviations are used in this manuscript:

Ti	Titanium
CoCrMo	Cobalt–chromium–molybdenum alloy
TiO_2_	Titanium dioxide
Mn	Manganese
N	Nitrogen
Si	Silicone
OCP	Open-circuit potential
V	Vanadium
P	Phosphorus
Fe	Iron
Al	Aluminum
W	Tungsten
EIS	Electrochemical impedance spectroscopy

## References

[1] Eichner K. (1983). Applications of metal alloys in dentistry—A review. Int. Dent. J..

[2] Xi D., Wong L. (2021). Titanium and implantology: A review in dentistry. J. Biol. Regul. Homeost. Agents.

[3] Lee J.J., Song K.Y., Ahn S.G., Choi J.Y., Seo J.M., Park J.M. (2015). Evaluation of effect of galvanic corrosion between nickel-chromium metal and titanium on ion release and cell toxicity. J. Adv. Prosthodont..

[4] Taher N.M., Al Jabab A.S. (2003). Galvanic corrosion behavior of implant suprastructure dental alloys. Dent. Mater..

[5] Chen W.Q., Zhang S.M., Qiu J. (2020). Surface analysis and corrosion behavior of pure titanium under fluoride exposure. J. Prosthet. Dent..

[6] Soares F.M.S., Elias C.N., Monteiro E.S., Coimbra M.E.R., Santana A.I.C. (2021). Galvanic corrosion of ti dental implants coupled to cocrmo prosthetic component. Int. J. Biomater..

[7] Amine M., Merdma W., El Boussiri K. (2022). Electrogalvanism in oral implantology: A systematic review. Int. J. Dent..

[8] Gittens R.A., Olivares-Navarrete R., Tannenbaum R., Boyan B.D., Schwartz Z. (2011). Electrical implications of corrosion for osseointegration of titanium implants. J. Dent. Res..

[9] Kim S.M. (2023). Oral galvanism related to dental implants. Maxillofac. Plast. Reconstr. Surg..

[10] Swalsky A., Noumbissi S.S., Wiedemann T.G. (2024). The systemic and local interactions related to titanium implant corrosion and hypersensitivity reactions: A narrative review of the literature. Int. J. Implant Dent..

[11] Schmalz G., Garhammer P. (2002). Biological interactions of dental cast alloys with oral tissues. Dent. Mater..

[12] Berbel L.O., Banczek E.D.P., Karoussis I.K., Kotsakis G.A., Costa I. (2019). Determinants of corrosion resistance of Ti-6Al-4V alloy dental implants in an in vitro model of peri-implant inflammation. PLoS ONE.

[13] Tagger Green N., Machtei E.E., Horwitz J., Peled M. (2002). Fracture of dental implants: Literature review and report of a case. Implant Dent..

[14] Ibrahim A.M.H., Takacova M., Jelenska L., Csaderova L., Balog M., Kopacek J., Svastova E., Krizik P. (2021). The effect of surface modification of timg composite on the in-vitro degradation response, cell survival, adhesion, and proliferation. Mater. Sci. Eng. C Mater. Biol. Appl..

[15] Mellado-Valero A., Muñoz A.I., Pina V.G., Sola-Ruiz M.F. (2018). Electrochemical behaviour and galvanic effects of titanium implants coupled to metallic suprastructures in artificial saliva. Materials.

[16] (2019). Dentistry—Compatibility Testing for Metal-Ceramic and Ceramic-Ceramic Systems.

[17] (2022). Dentistry—Metallic Materials for Fixed and Removable Restorations and Appliances.

[18] Procházková J., Podzimek S., Tomka M., Kucerová H., Mihaljevic M., Hána K., Miksovský M., Sterzl I., Vinsová J. (2006). Metal alloys in the oral cavity as a cause of oral discomfort in sensitive patients. Neuro Endocrinol. Lett..

[19] Venugopalan R., Lucas L.C. (1998). Evaluation of restorative and implant alloys galvanically coupled to titanium. Dent. Mater..

[20] Tuna S.H., Pekmez N.O., Keyf F., Canli F. (2009). The electrochemical properties of four dental casting suprastructure alloys coupled with titanium implants. J. Appl. Oral Sci..

[21] Fischer J. (2000). Mechanical, thermal, and chemical analyses of the binary system Au-Ti in the development of a dental alloy. J. Biomed. Mater. Res..

[22] Stanec Z., Halambek J., Maldini K., Balog M., Križik P., Schauperl Z., Ćatić A. (2016). Titanium ions release from an innovative titanium-magnesium composite: An in vitro study. Acta Stomatol. Croat..

[23] Tahmasbi S., Ghorbani M., Masudrad M. (2015). Galvanic corrosion of and ion release from various orthodontic brackets and wires in a fluoride-containing mouthwash. J. Dent. Res. Dent. Clin. Dent. Prospect..

[24] Klok O., Igual Munoz A., Mischler S. (2020). An overview of serum albumin interactions with biomedical alloys. Materials.

[25] Royhman D., Radhakrishnan R., Yuan J.C., Mathew M.T., Mercuri L.G., Sukotjo C. (2014). An electrochemical investigation of TMJ implant metal alloys in an artificial joint fluid environment: The influence of pH variation. J. Craniomaxillofac. Surg..

[26] Slama H., Cheniti H., Azem S., Nouveau C., Alhussein A. (2025). electrochemical evaluation of ti-based high-entropy alloys in artificial saliva with fluoride. Materials.

[27] Vishnu J., Kesavan P., Shankar B., Dembińska K., Swiontek Brzezinska M., Kaczmarek-Szczepańska B. (2023). Engineering antioxidant surfaces for titanium-based metallic biomaterials. J. Funct. Biomater..

[28] Turkina A.Y., Makeeva I., Dubinin O., Polyakova M., Sokhova I., Babina K., Zaytsev A., Dormidontova A. (2023). The impact of commercially available dry mouth products on the corrosion resistance of common dental alloys. Materials.

[29] Teixeira J.V.U., Pintão C.A.F., Correa D.R.N., Grandini C.R. (2023). Ti-15Zr and Ti-15Zr-5Mo Biomaterials alloys: An analysis of corrosion and tribocorrosion behavior in phosphate-buffered saline solution. Materials.

[30] Bokobza L. (2024). On the Use of Nanoparticles in Dental Implants. Materials.

[31] Shemtov-Yona K., Miara Y., Rittel D. (2025). Investigating the integrity of titanium-oxide nanolayers of Ti6Al4V under chemo-mechanical stress. Dent. Mater..

[32] Pellegrini G., Francetti L., Barbaro B., Del Fabbro M. (2018). Novel surfaces and osseointegration in implant dentistry. J. Investig. Clin. Dent..

[33] Igual-Munoz A., Genilloud J.-L., Jolles B.M., Mischler S. (2023). Influence of different sterilization methods on the surface chemistry and electrochemical behavior of biomedical alloys. Bioengineering.

[34] Javadi S., Castro L., Arrabal R., Matykina E. (2025). Metal ion release from PEO-coated Ti6Al4V DMLS alloy for orthopedic implants. J. Funct. Biomater..

[35] Chaturvedi T.P. (2009). An overview of the corrosion aspect of dental implants (titanium and its alloys). Indian J. Dent. Res..

[36] Yilmazer H., Caha I., Dikici B., Toptan F., Isik M., Niinomi M., Nakai M., Alves A.C. (2023). Investigation of the influence of high-pressure torsion and solution treatment on corrosion and tribocorrosion behavior of CoCrMo alloys for biomedical applications. Crystals.

[37] Guindy J.S., Schiel H., Schmidli F., Wirz J. (2004). Corrosion at the marginal gap of implant-supported suprastructures and implant failure. Int. J. Oral Maxillofac. Implant..

[38] Van Loven K., Jacobs R., Swinnen A., Van Huffel S., Van Hees J., van Steenberghe D. (2000). Sensations and Trigeminal somatosensory-evoked potentials elicited by electrical stimulation of endosseous oral implants in humans. Arch. Oral Biol..

[39] Martin M.D., Broughton S., Drangsholt M. (2003). Oral lichen planus and dental materials: A case-control study. Contact Dermat..

